# Brugada Syndrome and Pregnancy: Highlights on Antenatal and Prenatal Management

**DOI:** 10.1155/2014/531648

**Published:** 2014-05-22

**Authors:** Laura Giambanco, Domenico Incandela, Antonio Maiorana, Walter Alio, Luigi Alio

**Affiliations:** Obstetric and Gynecology Department, Civico Hospital, Piazza N. Leotta 4, 90100 Palermo, Italy

## Abstract

*Introduction*. Brugada syndrome is characterized by a disruption of heart's normal rhythm. It is an autosomal dominant disease due to a mutation of SNC5A gene. Its prevalence is low all over the world, but it is a lethal disease. Sudden cardiac death is the result of phenotypic manifestation of Brugada syndrome. Among asymptomatic Brugada patients, arrhythmia could be provoked by physical activity, fever, or pregnancy. About obstetrical management, very few data or reports have been published since this syndrome has been diagnosed in late 1992. * Case Presentation*. A 20-year-old pregnant woman at 13 weeks of gestation was referred to our department because of her familial history of sudden cardiac deaths. Brothers and sisters of her mother died of Brugada syndrome in childhood or older and live components of this family were carrier of mutation in Brugada gene. The pregnancy was uneventful. The patient gave birth vaginally without any arrhythmia. Strictly cardiological monitoring was performed during labour, delivery, and 12 hours of the postpartum. * Conclusion*. Even though patient at low risk may never have arrhythmia, some conditions could represent a Brugada trigger. The management could be very easy and uneventful. Otherwise it could be very difficult with need of ECMO or antiarrhythmics drugs or intracardiac device. Obstetrical management of Brugada pregnant women should be very strict and multidisciplinary in cooperation with cardiologist and anaesthesiologist and should provide an informed consent to the couple.

## 1. Background

Brugada syndrome is characterized by dysfunction of heart's normal rhythm. This disorder could lead to uncoordinated ventricular electrical activity (ventricular arrhythmia, syncope, sudden cardiac death). Symptomatic patients have typical ECG signs: ST segment elevation in right precordial without demonstrable structural heart disease [1, 2]. Brugada syndrome is an autosomal dominant disease.

The first genic mutation was recognized in SNC5A, gene involved in functioning sodium channels [3]. Indeed only 15%–30% of Brugada families have this mutation. Further sodium/calcium channel mutations have been identified in some genes: SNC1B, CACNA1C, CACNB2, and GDP1L [3]. Patients with Brugada syndrome have normal heart function but are at risk of cardiac arrhythmia; thus, the syndrome is one of the leading causes of death for young men in Southeast Asia [4]. The only effective prevention of sudden death is implantable cardioverter defibrillators (ICD) [3, 4], but it is not still clear which patients should be treated.

The sex-related difference in the phenotypic expression of the Brugada syndrome is more pronounced than in any other autosomally transmitted arrhythmic syndrome. The basis for this intriguing sex-related distinction is not fully understood.

Potential explanations are gender-related intrinsic differences in ionic currents and hormonal influences. Due to this hormonal influence, pregnancy represents a particular situation in the life of women with BS. To date, data elucidating the role of hormonal changes secondary to pregnancy in the clinical outcome of this population have been missing [5].

It is possible to subdivide patients as symptomatic, asymptomatic, positive, or negative to drugs test and member of symptomatic or asymptomatic family. The management, the risk stratification, and the quality of life are variable for each category. Nowadays Brugada syndrome is endemic in Southeast Asia and is increasing in Europe and in USA, but it still remains a rare disease with a prevalence estimated of 5–50 cases/10.000 [6]. Males have higher disease prevalence in adulthood (prevalence ratio of males to women 8 : 1), but gender difference is not relevant during childhood [7, 8]. Estradiol causes an increase of the inflow current that would result in antiarrythmic effect in Brugada syndrome's people [9]. There are very few data published on pregnancy, delivery modalities, and postpartum in Brugada women [5, 10].

## 2. Case Presentation

A 20-year-old nulliparas woman was referred to risky-pregnancy unit of our department. She had a familial history of sudden cardiac death for Brugada syndrome. Her brother died at 12 months for sudden infant death syndrome (SIDS). In her mother's family, another brother and three cousins died in childhood suddenly. One of the latter was temporarly resuscitated (even in anoxic coma) and a Brugada diagnosis was made on the basis of electrocardiographic pattern. Another aunt died older of Brugada syndrome even though she had implanted an internal cardiac defibrillator. In live components of this family and in our pregnant patient, a gene mutation was found out in SCN5A gene: genic substitutive mutation L567Q. The Brugada pregnant woman has been always asymptomatic during her life. Her basal electrocardiogram was normal as the QT was. In her childhood, our patient underwent drug provoking test (using flecainide and isoproterenol) with negative results.

So by risk stratification, she had a low risk for Brugada symptoms. Nonetheless, an internal cardiac defibrillator was implanted to her twice. The first device was recalled by the producer brand and the second one was removed because of inappropriate releasing electrical charge. At the age of 14, the patient and her parents decided not to substitute the second device with a new one. She kept asymptomatic and was referred to a tertiary care center more for familial history rather than for her symptoms. At first appointment, she was at 13 weeks of pregnancy. Hematologic values, ECG pattern, and ultrasonographic findings were found normal. Her pregnancy was uneventful during all gestation. She was informed about the theatrical risk of becoming symptomatic and about the inheritance of gene mutation.

## 3. Outcome and Follow-Up

A multidisciplinary team (senior obstetrician, cardiologist, neonatologist, and anesthesiologist) planned labour induction at 39,5 weeks to induce labor by endovaginal prostaglandins (1 mg). The scheduled admission was scheduled in order to have a senior obstetrical and cardiologist team available until she delivered. Active phase of labor started after 6 hours of prostaglandin's induction and she gave birth vaginally after 2 hours more a 3340-gram baby boy. During postpartum period, no arrhythmia appeared such as in pregnancy and in delivery. The patient's cardiac rhythm was monitored continuously during second and third stage of labor, postpartum till 12 hours after delivery. She breastfed her baby and was discharged 48 hours after vaginal delivery. She is still asymptomatic and her child is a 1-year-old Brugada patient (low risk).

## 4. Discussion

Some reports suggested that pregnancy could represent a trigger for arrhythmia [7] probably due to hemodynamic and hormonal changes. Indeed we want to remind that estrogens seem having a protective effect on Brugada syndrome or on its phenotype. Furthermore the male gender prevalence is established. Among affected people, Brugada is more clinical aggressive in male than females [11]. The role of hormonal changes during pregnancy and postpartum has not been evaluated. Nonetheless, Sharif-Kazemi et al. [5] reported an electrical storm during pregnancy as first sign of Brugada syndrome. Pagel et al. [12] in 2009 proposed the use of extracorporeal membrane oxygenation (ECMO) in order to Stabilize circulatory instability during incessant ventricular fibrillation in a pregnant patient with Brugada syndrome. Fever due to mastitis in postpartum period could unmask this syndrome [13], as reported in a woman who gave birth without any arrhythmia. Based on few literature data, Brugada's women may have normal pregnancies and may gave birth vaginally or by caesarean section without any electrical storm [5, 10, 13]. A risk stratification of Brugada women both for obstetrical and perinatal outcome is mandatory. Nowadays it is well established that Brugada patients have a great phenotypic variability. They could range from asymptomatic form to sudden cardiac death. Silent carriers of mutation may never exhibit disease's symptoms [4] or could reveal their illness due to a trigger (fever, physical activity, and pregnancy). How to and if manage those asymptomatics patients is still controversial. Many temptative of stratifying the risk have been performed with patient registry [4]. One of these registries revealed that 8% of initially asymptomatic patients died or had ventricular fibrillation during follow-up. The strongest predictive risk was a positive invasive electrophysiological study. A second research published [4] demonstrated that inducibility at electrophysiology study is nonpredictive of clinical aggressivity of disease. Lastly, other authors [14] underlined that asymptomatic Brugada patients with normal ECG baseline not responding to drug test (with ajmaline, flecainide, and procainamide) could live healthy and uneventful and could not require any treatment. As Brugada syndrome has rather low prevalence (1 : 2000) (depending on gender and geographic area), many clinical and critical characteristics are not well known by many physicians. It has been demonstrated that women can continue a pregnancy, can give birth vaginally, and can breastfeed in asymptomatic way or with arrhythmic disorders. It is mandatory to be aware, when managing Brugada patients, what kind of drugs should be avoided [15] (Table 1) and what kind of anesthesia is possible to perform. However obstetrical management of Brugada pregnant women should be multidisciplinary in cooperation with cardiologist and anaesthesiologist and should provide an informed consent to the couple (both for maternal and neonatal outcome).

## Figures and Tables

**Table 1:** Analgesic/anesthetic agent forbidden in Brugada patient (mod, 14).

Generic name	Clinical use/class	References	Recommendation
Bupivacaine	Analgesic agent/anesthetic	De La Coussave 1992 [16], Berman 1994 [17], Phillips 2003 [18], Vernogy 2006 [19], Bramall et al. 2011 [10]	IIa

Procaine	Analgesic agent	Arumugam 2012 [20]	IIa

Propofol	Anestehtic agent	Saint 1998 [21], Inamura 2006 [22], Vernogy 2006 [19], Robinson 2008 [23]	IIb

Leg: Class IIa: There is conflicting evidence and/or divergence of opinion about the drug, but the weight of evidence/opinion is in favor of a potentially arrhythmic effect in Brugada syndrome patients.

Class IIb: There is conflicting evidence and/or divergence of opinion about the drug, and the potential arrhythmic effect in Brugada syndrome patients is less well established by evidence/opinion.
